# Enhanced thrombolysis by endovascular low-frequency ultrasound with bifunctional microbubbles in venous thrombosis: *in vitro* and *in vivo* study

**DOI:** 10.3389/fbioe.2022.965769

**Published:** 2022-07-22

**Authors:** Zhaojian Wang, Yunfan Pan, Huaigu Huang, Yuan Zhang, Yan Li, Chenghong Zou, Guanghua Huang, Yuexin Chen, Yongjian Li, Jiang Li, Haosheng Chen

**Affiliations:** ^1^ Department of Vascular Surgery, Peking Union Medical College Hospital, Chinese Academy of Medical Sciences and Peking Union Medical College, Beijing, China; ^2^ State Key Laboratory of Tribology, Tsinghua University, Beijing, China; ^3^ School of Mechanical Engineering, University of Science and Technology Beijing, Beijing, China

**Keywords:** low-frequency ultrasound (LFUS), sonothrombolysis, microbubbles, CREKA, venous thrombus

## Abstract

There is a need to improve the efficacy and safety of endovascular techniques in venous thrombotic diseases, and microbubble enhanced sonothrombolysis is a promising approach. However, whether endovascular low-frequency ultrasound (LFUS) can be utilized in microbubble enhanced sonothrombolysis is unclear. Here, we present a catheter-based thrombolytic system that combines unfocused low-frequency low-intensity ultrasound with novel fibrin-targeted drug-loaded bifunctional microbubbles. We develop an *in vitro* flow model and an *in vivo* rabbit inferior vena cava (IVC) thrombosis model to evaluate the safety and efficacy of the thrombolytic system. The results indicate that microbubble enhanced sonothrombolysis with endovascular LFUS treatment for 30 min is equally effective compared to pure pharmacologic treatment. Furthermore, the thrombolytic efficacy of this system is safely and substantially improved by the introduction of a fibrin-targeted drug-loaded bifunctional microbubble with a reduction of the fibrinolytic agent dosage by 60%. The microbubble enhanced endovascular LFUS sonothrombolysis system with excellent thrombolytic efficacy may serve as a new therapeutic approach for venous thrombotic diseases.

## Introduction

Recent advances in endovascular pharmacomechanical interventions allow early removal of thrombus in venous thromboembolism (VTE). However, the current devices face challenges like risk of bleeding, residual thrombus, and post-thrombotic syndrome (PTS) (Higuma et al., 2016; Broderick et al., 2021). Therefore, creation of an efficient, site-specific, safe endovascular approach as an adjuvant or alternative therapy is still in urgent need.

In recent years, microbubble enhanced sonothrombolysis has emerged as a promising therapeutic method for thrombotic diseases, and is endowed with the merits of ultrasound (US) and microbubbles (safety, ease of use, cost-effectiveness, and clinical availability) (Ilovitsh et al., 2020; El Kadi et al., 2022). Extensive researches indicate that ultrasound and microbubbles may enhance thrombolysis through the mechanism of acoustic radiation, cavitation-mediated microstreaming, and microjet, facilitating enzymatic degradation and mechanical destruction of thrombus (Goel and Jiang, 2020). One of the most exciting advantages the treatment modality prospects is the dosage reduction of thrombolytic agents, which may be critical for those high bleeding risk patients in which the use of thrombolytic agents is contradicted.

Currently, the delivery of ultrasound through a catheter-based endovascular approach has become the mainstream choice in sonothrombolysis. To date, several endovascular ultrasonic catheters which adopt high-frequency (620 kHz-2.2 MHz) low-to-moderate intensity (0.5–11 W/cm^2^) ultrasound have been developed (Kim et al., 2017; Engelberger et al., 2019; Li et al., 2021). However, all these catheters chose a relatively high frequency of ultrasound from sub-megahertz to several megahertz, leaving the thrombolytic effect of ultrasound below 100 kHz largely unexplored. In addition, the use of high frequency ultrasound is restricted by the potential thermal issue. Prolonged exposure time with high frequency US results in increased heat absorption in the collateral tissue (Li et al., 2021). A cooling system and a restricted duty cycle (usually < 10%) are usually deployed to avoid potential thermal damage during the treatment, which inevitably compromises the thrombolytic efficacy (Schäfer et al., 2005).

Evidence from externally-delivered ultrasound has suggested that the ultrasound with a lower frequency not only has a higher thrombolytic efficacy but also has a lower thermal effect and deeper thrombus penetration (Chowdhury et al., 2020). In theory, low-frequency ultrasound (LFUS), usually defined as a frequency between 20 and 100 kHz, (Ahmadi et al., 2012), has several advantages for thrombolysis. First, the enhancement of MB oscillations facilitates cavitation-related mechanical effects. Observations in several studies have indicated that high-amplitude MB oscillations were excited by LFUS at a relatively low peak negative pressure (Ilovitsh et al., 2020). This will lead to increased exposure of new binding sites for plasmin in the fibrin mesh, continuous removal of fibrin degradation products in a micropump-like behavior, and enhanced pressure waves exerting on the thrombus, thereby accelerating the clot dissolution (Datta et al., 2006). Second, the use of LFUS enlarges the focal zone and increases the penetration depth, covering a larger area in the treatment of extensive thrombi (Ammi et al., 2015). In addition, LFUS has lower tissue attenuation, and a lower risk of thermal damage, which may save the need for an intraoperative coolant (Suchkova et al., 2002). Based on previous externally applied ultrasound, *in vitro* and *in vivo* studies have confirmed that the external LFUS generated by transducers at several centimeters distance is efficient for accelerating enzymatic thrombolysis within a relatively low range of intensities (0.25–3 W/cm^2^) (Suchkova et al., 1998; Siegel et al., 2000; Suchkova et al., 2000). Although the incremental effect in the thrombolytic efficacy by external LFUS has been confirmed, whether this effect applies to the endovascular setting with microbubbles is not clear. In addition, few studies have reported the *in vivo* results of MB enhanced endovascular sonothrombolysis.

In this study, we investigate the thrombolytic effect of endovascular LFUS with microbubbles in the flow model *in vitro* and the rabbit IVC thrombosis model *in vivo*. To achieve this, a unique needle probe-tipped ultrasonic catheter was designed to deliver LFUS and microbubbles. A novel fibrin-targeted drug-loaded bifunctional MB was introduced to further improve the thrombolytic efficacy. We propose that combined microbubbles and endovascular LFUS effectively dissolve venous thrombus, and LFUS-mediated sonothrombolysis can be safely and effectively promoted by the fibrin-targeted drug-loaded bifunctional MBs, with a significant dosage reduction of the fibrinolytic agent. Our study may promote the application of LFUS in endovascular microbubble enhanced sonothrombolysis therapy, and advance current treatment with higher thrombolytic efficacy to achieve early recanalization in VTE.

## Results and discussion

### Endovascular microbubble-enhanced low-frequency ultrasound sonothrombolysis system and the experimental platform

**FIGURE 1:**
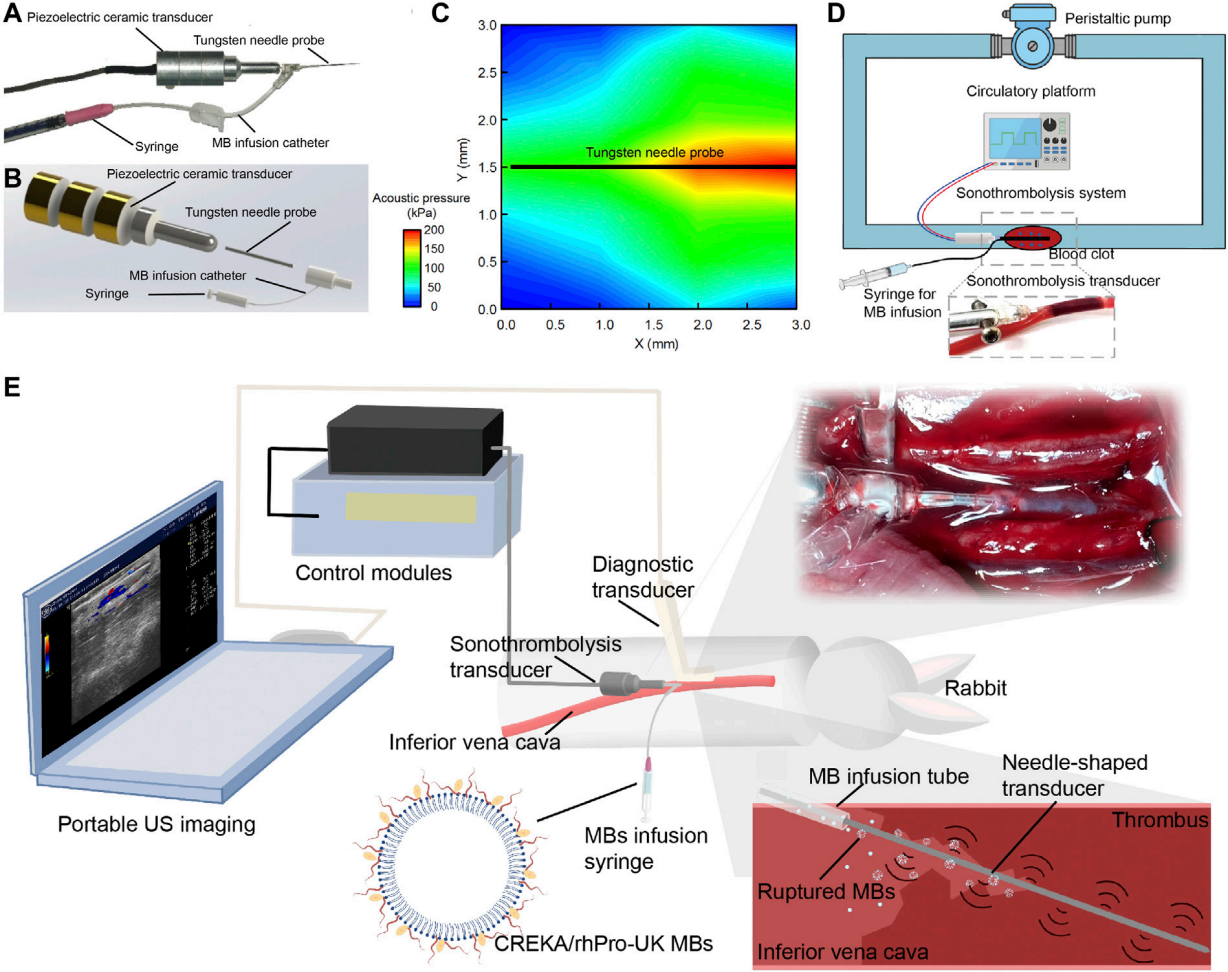
Endovascular microbubble-enhanced LFUS sonothrombolysis system and the experimental platform. **(A)** Physical display and **(B)** schematic display of the endovascular microbubble-enhanced sonothrombolysis system. The tungsten needle probe is connected to piezoelectric ceramic transducer. The intra-clot delivery of LFUS is realized through the insertion of needle probe into the blood clot. At the end of the needle probe is a catheter for MB delivery. **(C)** The spatial distribution of acoustic pressure adjacent to the tungsten needle probe. **(D)** Schematic display of the flow model *in vitro*. A magnified image of the needle probe inserting into the tube and blood clot is presented. **(E)** Schematic display of rabbit IVC thrombosis model and microbubble-enhanced sonothrombolysis systems with a portable US imaging device for intraoperative ultrasonic evaluation. A magnified image and a schematic display of the needle probe inserting into the IVC thrombosed segament is presented.

An endovascular microbubble-enhanced LFUS sonothrombolysis system in accord with safety standards was designed. As a proof-of-concept design to verify our hypothesis on the thrombolytic effect of LFUS + MBs, this prototype system consists of four modules (Figures 1A,B): 1) the control module, which connects to a power source and regulates the ultrasonic energy output parameters; 2) the external piezoelectric transducer that generates and transmits the ultrasonic energy to the needle probe; 3) the tungsten needle probe that inserts into the thrombus to perform sonothrombolysis (0.6 mm in diameter, 30.0 mm in length); 4) the catheter at the end portion of needle probe that delivers MBs direct to the treatment zone. The input voltage is 30 V_pp_. The intensity (I_SPTA_) is 2.58 W/cm^2^, which is within the maximum effective intensity limit of 3 W/cm^2^ by the International Electrotechnical Commission (Ahmadi et al., 2012). The ultrasound impulse emits in a continuous mode at a working frequency of 47.1 kHz. The needle hydrophone test confirms an unfocused acoustic field whose energy peaks at the tip of the transducer (Figure 1C). The peak negative pressure is 212 kPa. Mechanical Index (MI), defined as peak negative pressure (in MPa) divided by the square root of the operating frequency (in MHz), is calculated to be approximately 0.98, which is within the FDA limit of 1.9 (Ahmadi et al., 2012).

Previous research has shown that the thrombolytic efficiency of ultrasound depends directly on use of MBs, intensity, duration, and inversely on frequency (Suchkova et al., 2002; Schäfer et al., 2005; El Kadi et al., 2022). The design rationale of our device is that combined advantages of LFUS, microbubble, and continuous mode may result in higher thrombolytic efficacy and reduce the dependence on thrombolytic agents. Our experiment has confirmed by monitoring thermal damage in the safety evaluation that endovascular sonothrombolysis system is allowed to deliver LFUS in a continuous mode (100% of duty cycle) without raising the regional temperature, which potentially serves as an advantage over other devices by ensuring sufficient ultrasonic exposure during the treatment.

Unlike resting fluid systems in previous endovascular thrombolysis tests, (Kim et al., 2017; Engelberger et al., 2019; Li et al., 2021), our *in vitro* flow model in this study allows simulation of physiological or pathological flows, thus creating the mechanical stress and continuous replenishment of substances important to thrombolysis (Greenberg et al., 2000). The flow rate is 20 ml/min with temperature maintained at 37 ± 0.3°C. During the treatment, a total of 2.7 casein plasminogen was loaded to flow model at the beginning of thrombolysis to simulate the endogenous plasminogen *in vivo*. (Figure 1D). In the rabbit IVC thrombosis model *in vivo*, the venous thrombus was confirmed by ultrasonic evaluation. Mean blood flow velocity (BFV) before and after the induction of IVC thrombus were 12.14 ± 3.43 cm/s, 0.20 ± 0.29 cm/s, respectively. The needle probe was inserted into the thrombus to perform sonothrombolysis (Figure 1E, Supplementary Figure S1).

### Thrombolytic efficacy of combined low-frequency ultrasound with MBs without thrombolytic agent *in vitro* and *in vivo*

In this part, we sought to validate the thrombolytic effect of continuous low-frequency low-intensity ultrasound in combination with local MBs. The thrombolytic efficacy of combined LFUS with MBs was evaluated in the flow model *in vitro* and in the rabbit IVC venous thrombosis model *in vivo*.

In the *in vitro* study, the wet weight of blood clot before (*w*_*0*_) and after treatment (*w*_*1*_) was determined to evaluate the thrombolytic efficacy. The mean weight of *ex vivo* blood clots was 0.22 ± 0.05 g before treatment. There was no significant difference in the mean weight of blood clots among the groups. Next, the blood clots were placed in the flow model *in vitro* and subjected to 30-min thrombolysis. Thrombolytic efficacy was evaluated by clot lytic ratio (LR, %) and velocity of clot lysis (VLR, %/min). The calculations are shown in the following Eq. 2, Δ*T* = 30 min:

$$LR=\left(\frac{w_{0}-w_{1}}{w_{0}}\right)\times 100\%\;\;$$ (1)

$$VLR=\frac{LR}{\Delta T}\;\;$$ (2)

**FIGURE 2:**
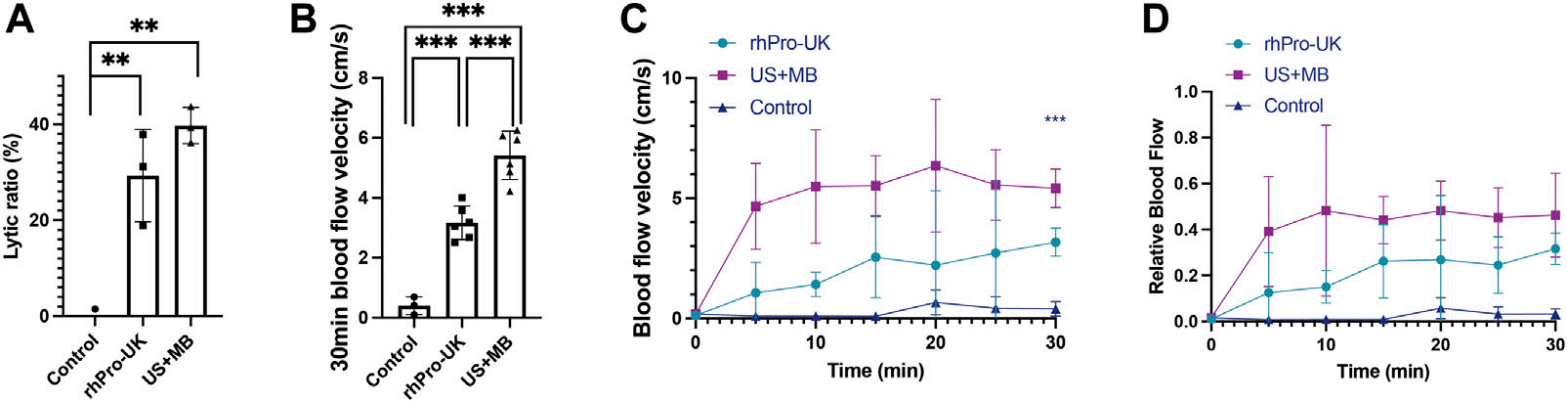
Microbubble enhanced endovascular LFUS sonothrombolysis. **(A)** Lytic ratio *in vitro*. **(B)** The 30-min blood flow velocity *in vivo*. **(C)** Blood flow velocity curves in 30 min treatment *in vivo.* **(D)** Relative blood flow compared to baseline in 30 min treatment *in vivo* (^***^ *p* < 0.001, ^**^ *p* < 0.01).

The clot lytic ratio was 39.7 ± 3.7% in the US + MB group, 29.3 ± 9.6% in the rhPro-UK group, 0.0 ± 1.5% in the control group (n = 3), suggesting that the combined MB with LFUS has comparable thrombolytic effect compared to rhPro-UK infusion. (*p* = 0.233) (Figure 2A) The velocity of clot lysis was calculated as 1.32%/min, which was higher than 0.98%/min in the rhPro-UK group.

In the *in vivo* study, a significant improvement in blood flow recovery after 30 min in the US + MB group compared with the rhPro-UK group was observed. (Figure 2B, 5.4 ± 0.8 cm/s vs 3.2 ± 0.6 cm/s, *p* < 0.001). There is a sharp increase of BFV in the first 5 min in US + MB treatment, which is quite different from the curve in the rhPro-UK group where a gradual recovery of blood flow is observed. (Figures 2C,D). The rapid recovery of blood flow in US + MB treatment is impressive as this can be a life-saving feature in medical emergencies. The superiority of US + MB treatment demonstrated *in vivo* could be attributed to enhanced penetration of endogenous fibrinolytic enzyme and fast removal of degradation products as a consequence of enhanced microstreaming and fluid mixing (Datta et al., 2008; El Kadi et al., 2022).

Thrombolytic efficacy of our device was compared with other endovascular high-frequency ultrasonic catheters in the literature. The results indicate that the VLR of our system is the highest among current endovascular ultrasonic catheters under similar US + MB protocols. (Table 1). This could be attributed to a lower cavitation threshold and enhanced oscillation amplitude of MBs under LFUS in comparison with megahertz ultrasound, which is supported by our in silico simulation of microbubble oscillation using the Marmottant model and the literature (Nedelmann et al., 2005; Ahmadi et al., 2012; Ilovitsh et al., 2018) (Supplementary Figure S2). As a result, the number and amplitude of microbubbles undergoing cavitation increased, generating stronger mechanical erosion and microstreaming on the thrombus. In addition, a wider range of the acoustic field is seen in LFUS for ultrasonic radiation, which possibly contributes to the enhanced thrombolysis (Ammi et al., 2015). The above results suggest that endovascular thrombolysis using low kilohertz US plus MBs without thrombolytic agents has a similar thrombolytic effect compared with the pure pharmacologic infusion. The thrombolytic efficacy of microbubble enhanced endovascular sonothrombolysis with LFUS may surpass the systemic thrombolytic administration *in vivo*.

**TABLE 1:** Characteristics of endovascular sonothrombolysis catheter and VLR.

	Frequency	Power (V_pp_)	MI	PNP (kPa)	Duty cycle (%)	I_SPTA_ (W/cm^2^)	Thrombolytic agents	MB (µg)	VLR (%/min)
Kim et al. (2017)	620 kHz	80	1.0	250	10	11	None	45	0.7
EKOS, Engelberger et al. (2019)	2.2 MHz	-	0.7	102–221	10	0.5–4.9	0.2 mg rt-PA	45	0.54
Li et al. (2021)	1.1 MHz	50–60	0.4	414	5	4.89	None	45	0.23
US + MB	47.1 kHz	30	0.98	212	100	2.58	None	45	1.32
US + crukMB							4 mg rhPro-UK		2.8

### Thrombolytic efficacy of low-frequency ultrasound enhanced by CREKA/rhPro-UK MBs *in vitro* and *in vivo*

To further improve the thrombolytic efficacy, we integrate a novel fibrin-targeted CREKA/rhPro-UK MB (crukMB) to endovascular LFUS delivery system. (Supplementary Figure S3). CREKA (Cys-Arg-Glu-Lys-Ala) is a pentapeptide with a high affinity to the major acellular component of venous thrombus, i.e., fibrin (Wang et al., 2021). Human recombinant prourokinase (rhPro-UK) is one of the new thrombolytics and induces fibrin-selective clot lysis (Hao et al., 2018). The CREKA peptide and rhPro-UK are attached to the MB surface through direct conjugation methods according to the previous study (Mu et al., 2009). No significant difference in the concentration, mean diameter and size distribution occurred after the conjugation. The average loading capacity of crukMBs for rhPro-UK was (6.54 ± 1.58) ×10^3^ U/ml. (Table 2, Supplementary Figure S3).

**TABLE 2:** Characterization of MBs and crukMBs.

MB type	Concentration (× 10^7^ MB/ml)	Mean diameter (μm)	Drug-loading capacity (× 10^4^ U/ml)
blank MB	3.87 ± 0.17	3.2 ± 0.13	N/A
crukMB	3.27 ± 0.08	3.7 ± 0.24	6.54 ± 1.58
*p*-value	0.164	0.412	N/A

**FIGURE 3:**
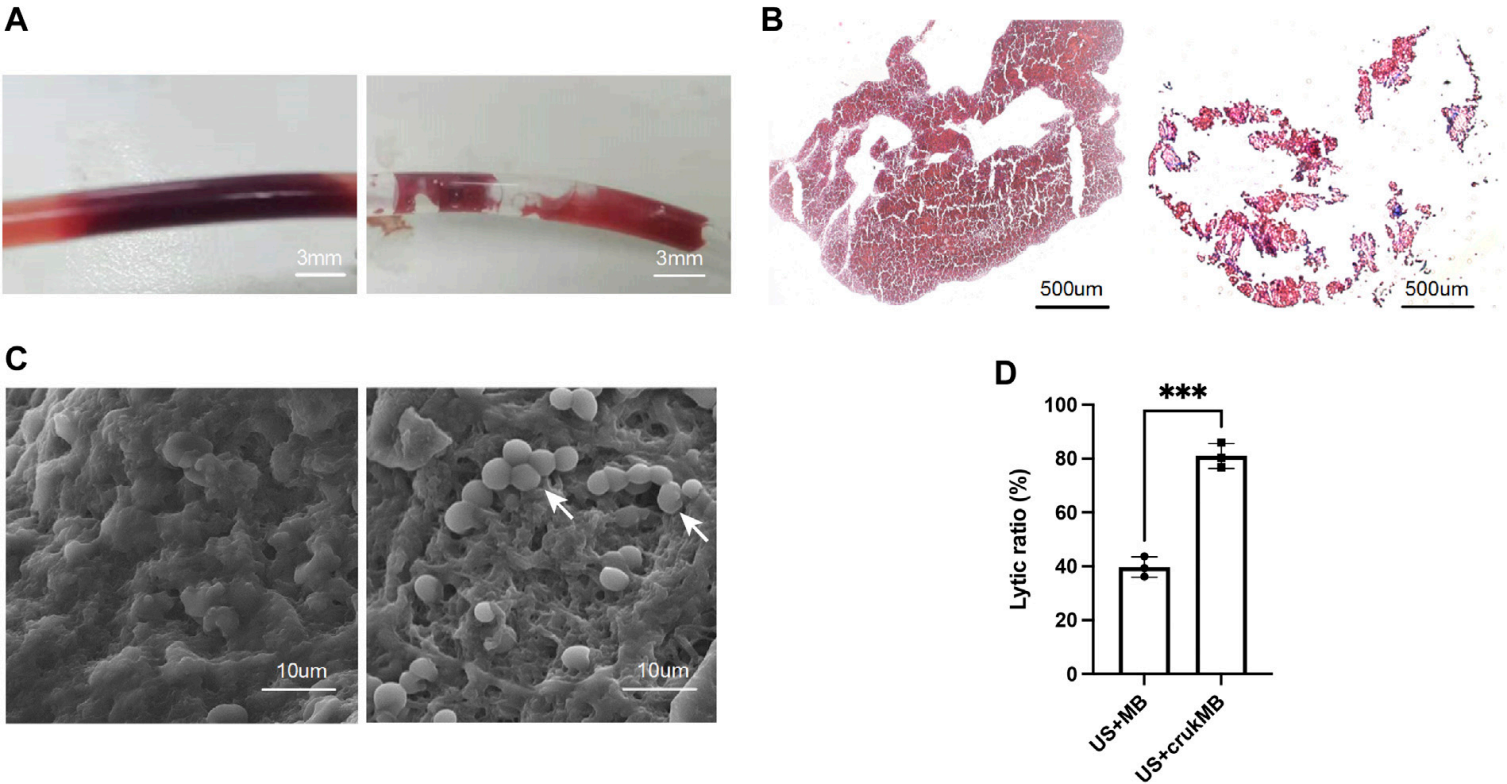
Enhanced sonothrombolysis with crukMBs *in vitro*. Blood clot images of macroscopy **(A)**, scale bars, 3 mm, bright field microscopy **(B)**, scale bars, 500 µm and SEM **(C)**, scale bars, 10 µm before and after LFUS + crukMB sonothrombolysis. White arrows indicate the exposed cellular components after fibrin degradation. **(D)** The 30-min lytic ratio of the US + MB group and US + crukMB group *in vitro*. (^***^ *p* < 0.001).

To investigate the effect of crukMBs on MB enhanced endovascular LFUS sonothrombolysis, thrombolytic efficacy of combined crukMBs plus LFUS was compared with control MBs plus LFUS *in vitro* and *in vivo*. In the *in vitro* thrombolysis test, blood clot images of macroscopy, bright field microscopy, and scanning electron microscopy before and after LFUS + crukMB thrombolysis are shown in Figures 3A–C. Macroscopic views of blood clots show a significant reduction in the clot burden after 30 min treatment, (Figure 3A), which is consistent with hematoxylin and eosin (H&E) staining under microscopy. (Figure 3B). In addition, scanning electron microscopy (SEM) confirms the thinned fibrin network and subsequent exposure of underlining cellular components after the LFUS + crukMB treatment, implying that crukMB may enhance thrombolytic efficacy through enzymatic and mechanical degradation of fibrin. (Figure 3C). The 30-min lytic ratio *in vitro* was 81.0 ± 4.7% in the US + crukMB group, significantly higher than 39.7 ± 3.8% in the US + MB group (*n* = 3, *p* < 0.001). (Figure 3D).

**FIGURE 4:**
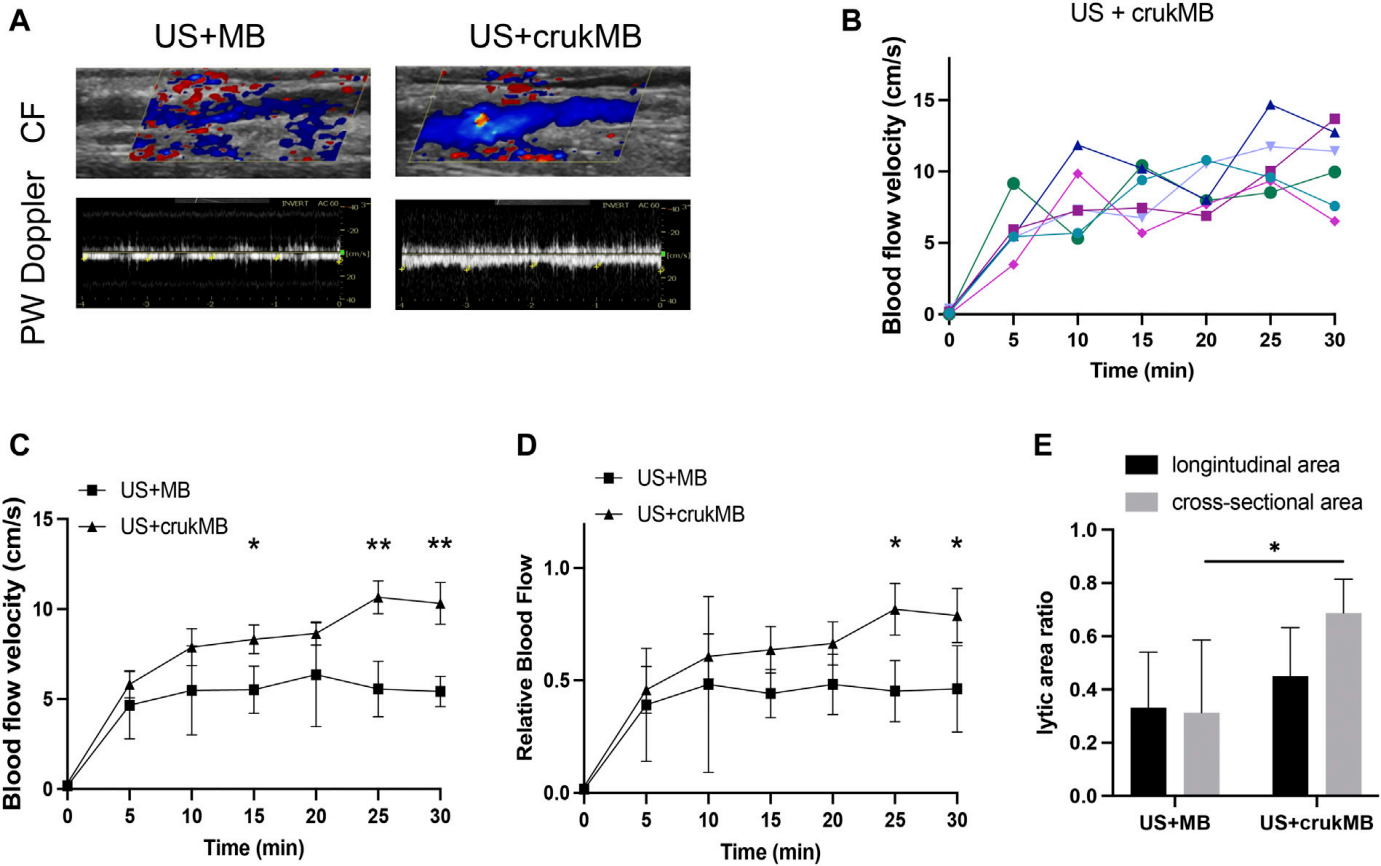
Enhanced sonothrombolysis with crukMB *in vivo*. **(A)** Ultrasonic evaluation of IVC thrombus after 30 min treatment. **(B)** Blood flow velocity curves in the US + crukMB group, each curve represents a treatment sample. **(C)** Blood flow velocity curves in 30 min treatment *in vivo.* **(D)** Relative blood flow compared to baseline in 30 min treatment *in vivo*. **(E)** 30-min lytic area ratio. (^***^ *p* < 0.001, ^**^ *p* < 0.01, ^*^ *p* < 0.05).

In the *in vivo* study, the ultrasonic evaluation was performed to evaluate thrombolysis and recanalization in the rabbit IVC thrombosis model. Restoration of blood flow was detected under color flow mode and pulse-wave mode. (Figure 4A). As shown in Figure 4B, each curve corresponds to the trend of blood flow velocity in each sample from the US + crukMB group. There was a significant increase in the extent of blood flow being restored (Figures 4C,D) and lytic rate indicated by the lytic area ratio. (Figure 4E). Targeted delivery and localized controlled release result in increased local drug bioavailability. With the help of bifunctional MBs, a lower drug concentration was achieved while still maintaining the efficacy of sonothrombolysis. Therefore, the risk of systemic bleeding decreases, and a broader population of patients can be included with this approach. (Ammi et al., 2015). The dosage of rhPro-UK carried by crukMB group (3.9 × 10^5^ U, calculated from Table 2) was only 40% of that in the rhPro-UK group (10.0 × 10^5^ U). With the LFUS, it is speculated that enhanced cavitation and synergistic effect of LFUS on enzymatic degradation accelerate sonothrombolysis (Wyshelesky et al., 2001). The above results suggest that the thrombolytic efficacy of LFUS + MB treatment can be further enhanced by fibrin-targeted rhPro-UK-loaded bifunctional crukMBs.

### Histological and safety evaluation

**FIGURE 5:**
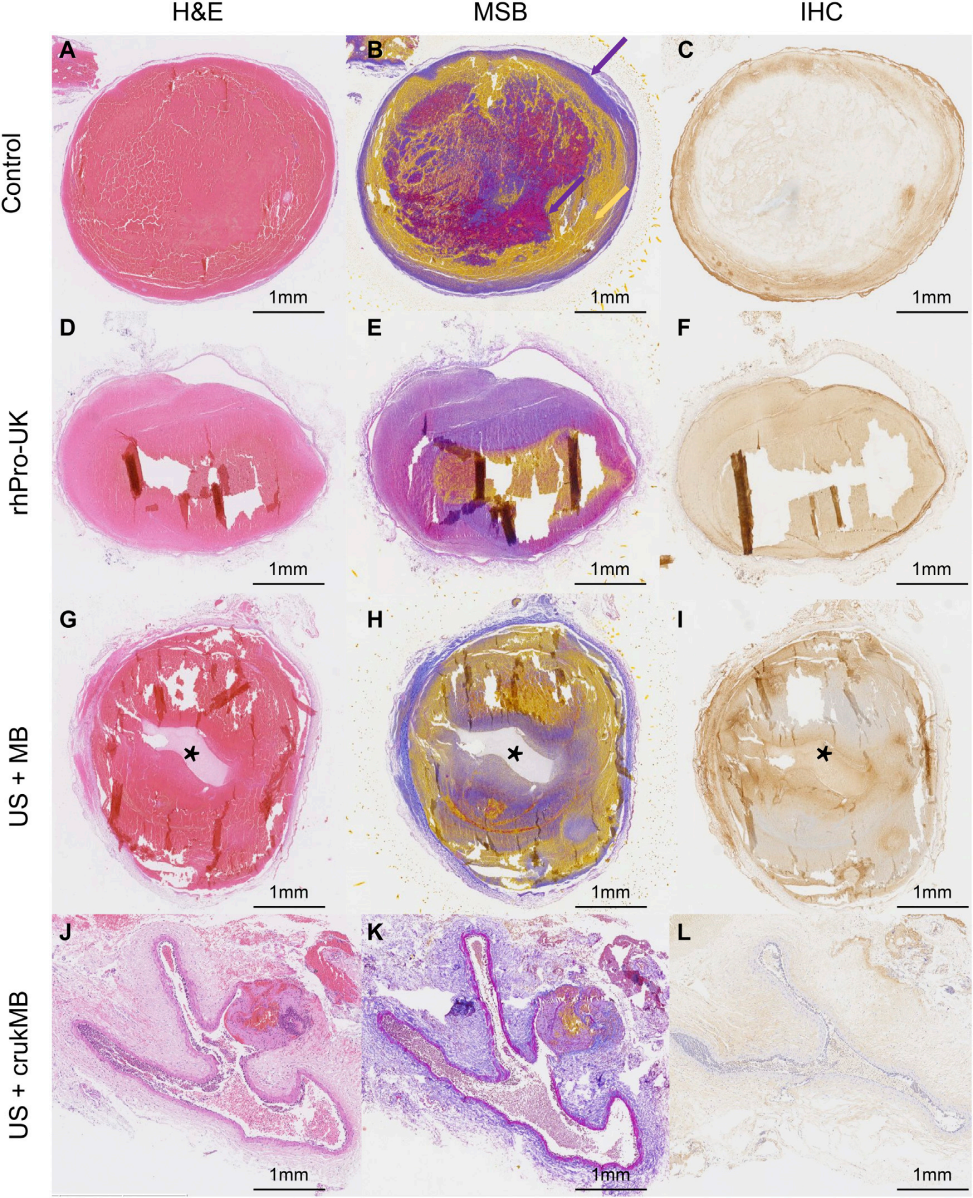
Histological analysis of post-treatment thrombus. Fibrin and red blood cells depositions were indicated by purple color and yellow color, respectively (purple- and yellow-colored arrows in panel **(B)**. The tunnels [asteroids in panels **(G–I)**] in the US + MB group were the results of treatment. The lumen of IVC in the US + crukMB group was collapsed as indicated in panels **(J-L)**, due to a nearly complete clearance of thrombus. (scale bars, 1 mm).

Microscopic histological analysis of post-treatment IVC thrombi with H&E, Martius Scarlet Blue (MSB), and anti-fibrin immunohistochemical (IHC) staining was performed to analyze the changes in thrombi. MSB staining identifies clot histological composition better than H&E staining. The results explain the variations in thrombolysis efficacy of different groups. As shown in Figures 5A–C, thrombus in the control group fully occupies the vessel lumen, resulting in a complete obstruction, hence no blood flow was observed in the experiment. In the rhPro-UK group, the hallow-like artifacts resulted from the detachment of clot fragments, indicating the loosened fibrous scaffold by fibrinolytic degradation of cross-linked fiber. (Figures 5D–F). The weak blood flow signal indicated in the experiment may result from fibrinolysis which leads to the formation of microtubules and micropores inside the thrombus. Sections from the US + MB group show that a central tunnel is formed in the pathway of the needle probe, and the diameter of the tunnel (1.5 mm) is much wider than that of the needle probe (0.6 mm), indicating fibrinolysis of the surrounding area. In addition, the hypo-chromatic region surrounding the tunnel in both MSB and IHC staining also suggests fibrin degradation (Figures 5G–I). This could be attributed to enhanced mechanical destruction and endogenous fibrinolysis under combined MB and ultrasound treatment. The effect of MB enhanced sonothrombolysis was most prominent in the US + crukMB group, evident by a significant reduction in the thrombus area and fibrin content. The IVC lumen naturally collapses with thrombus dissolved, showing the normal morphologic figure of a classic vein. (Figures 5J–L). As shown in the figure, recanalization was narrowly achieved in the US + MB group and was completely achieved in the US + crukMB group, which is in accord with the degree of blood flow recovery.

**FIGURE 6:**
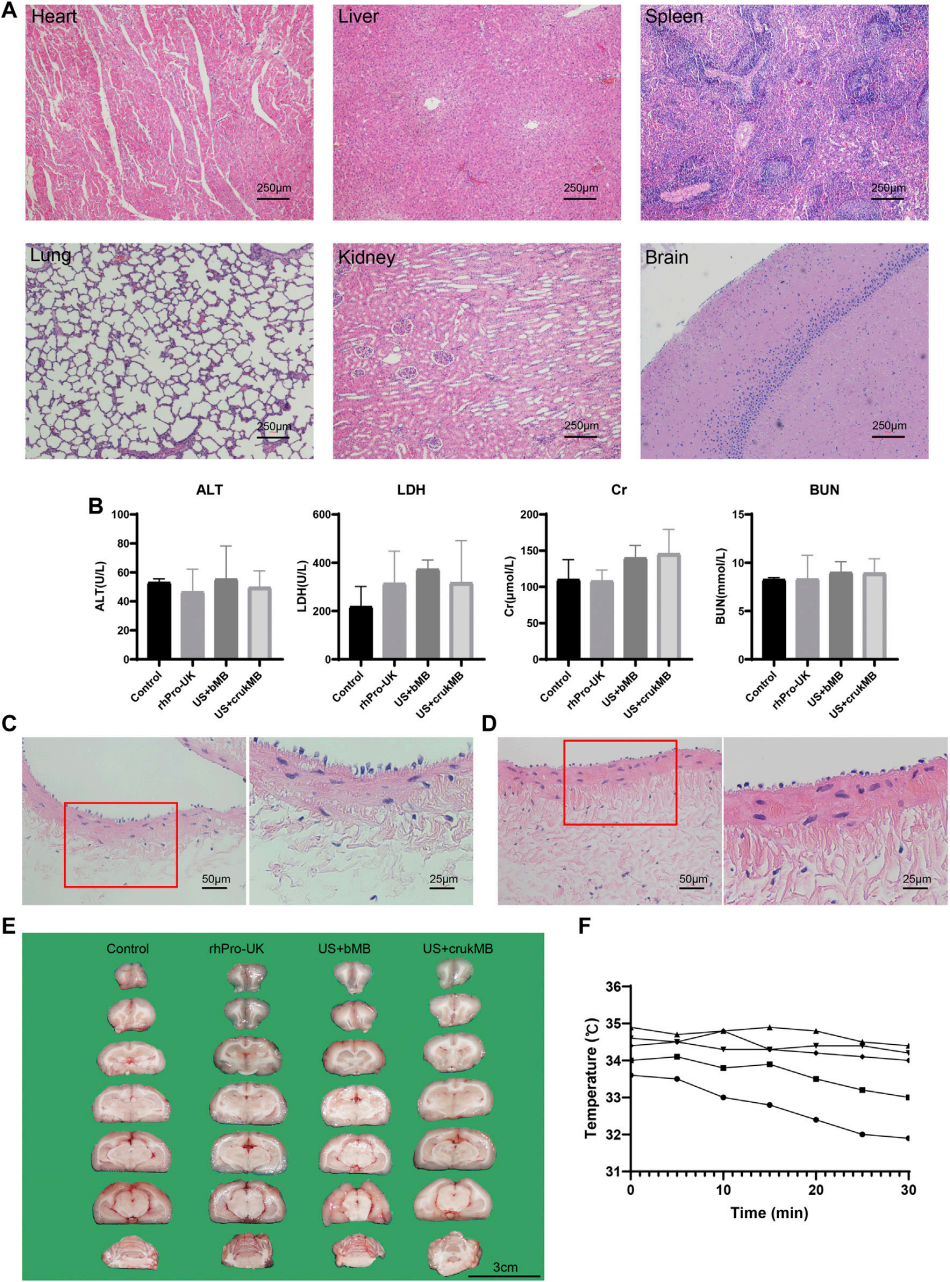
Safety evaluation. **(A)** Major organs (heart, liver, spleen, lung, kidney and brain) were harvsted for histological evaluation. No pathological founding was identified. (scale bars, 250 μm) **(B)** No difference was identified in the level of serum alanine aminotransferase (ALT), lactate dehydrogenase (LDH), creatinine (Cr), blood urea nitrogen (BUN). (*n* = 3–5) The lining of endothelia was comparable in vessels without **(C)** and with ultrasonic exposure **(D)**, respectively. (scale bars, 50 μm, 25 μm) **(E)** No hemorrhage was identified in the US + crukMB group. (scale bars, 3 cm; *n* = 3–6) **(F)** Temperature monitoring of during the 30-min sonothrombolysis treatment. Each curve represents an individual IVC model for sonothrombolysis treatment. No elevation of regional temperature during the treatment was observed. (*n* = 5).

Next, we investigated whether treatment with endovascular sonothrombolysis and the crukMBs had biosafety issues such as possible organ and tissue damage, unacceptable thermal effects, and hemolysis. No treatment-related organ damage was observed in the microscopic evaluation of major organs (Figure 6A and Supplementary Figure S4). There was no difference between the groups in postoperative biochemical markers (Figure 6B). No treatment-related endothelial injury was observed (Figures 6C,D). No hemorrhage was identified in the US + crukMB group. Intracerebral hemorrhages were only identified in two rabbits, one in the US + MB group and the other in the rhPro-UK group respectively (Figure 6E and Supplementary Figure S5, Supplementary Table S1). We did not observe the elevation of regional temperature in the treatment zone in the *in vivo* study under the experimental setting (Figure 6F).

## Conclusion

In this study, an endovascular microbubble enhanced LFUS sonothrombolysis system is successfully established. The low-frequency low-intensity endovascular ultrasound and microbubbles as a combined thrombolytic system is equally effective compared with the pure pharmacologic treatment. Furthermore, the thrombolytic efficacy of this combined system is safely and substantially improved by a fibrin-targeted drug-loaded microbubble with a reduction of fibrinolytic agents by 60%. Future directions of our study include refining the design and working parameters of endovascular LFUS delivery catheter based on the prototype in this study. Preclinical tests on large animals are needed to compare the system with current thrombectomy devices for both acute, subacute, and chronic thrombi. Our endovascular microbubble enhanced LFUS sonothrombolysis system with excellent thrombolytic efficacy may serve as a promising therapeutic approach for venous thromboembolism.

## Experiments

### Materials

All reagents were purchased from commercial suppliers and used as received without further modification. The CREKA, FITC-labeled CREKA, RGDS peptide were purchased from Qiangyao Biotechnology, China. The recombinant human prourokinase (rhPro-UK) was purchased from Tasly Biopharmaceuticals, China. The 5-TAMRA-labeled rhPro-UK was purchased from Nuofan Biotechnology, China. The Bradford protein assay kit was purchased from Beyotime Biotechnology, China. The anti-fibrinogen antibody (ab34269) was purchased from Abcam, United Kingdom. The MSB and H&E staining was purchased from Baso Diagnostics, China. The arbitrary function generator was purchased from AFG3101, Tektronix, United States. The needle hydrophone was from NCS-1, The Institute of Acoustics of the Chinese Academy of Sciences, China. The vortex shaker (DLAB MX-RD-E) was purchased from Medilab Tech, United States. Image Particle Size Analyzer (Pixtreme) was purchased from W2000M, United States. The microplate reader was purchased from Infinite, United States. The 96-well plate was purchased from Corning, United States. The bright-field microscope (IX83) was purchased from Olympus, Japan. The portable diagnostic US system (LOGIQ-e) was purchased from GE Healthcare, United States. The ImageJ software was from NIH, Maryland, United States. The thermometer was purchased from TM-902C, China. The automated analyzer (Dimension EXL-LM integrated chemistry system) was purchased from Siemens Healthcare Diagnostics Inc., Germany.

### Characterization of the endovascular low-frequency ultrasound transducer

The transducer was positioned in a tank filled with the degassed water. The transducer was driven by continuous cycle sinusoidal inputs at 47.1 kHz using an arbitrary function generator connected with a 12 dB radio-frequency amplifier. A needle hydrophone was used to measure the pressure output.

### Preparation and characterization of MBs

Commercially available phospholipid-encapsulated sulfur hexafluoride MBs were prepared according to manufacturer’s instruction. The preparation and characterization of fibrin-targeted CREKA/rhPro-UK MBs is described in the (Supplemental material).

### Thrombolysis *in vitro*

To test the thrombolytic effect of combined MBs and endovascular LFUS *in vitro*, an experimental platform was built with a flow model and mounted blood clot (Figure 1A). Briefly, *ex vivo* human whole blood clot was prepared *in vitro* by adding 1 ml of 75 mM CaCl_2_ and 35 mM MgCl_2_ to the 9 ml blood sample pretreated with 0.9 ml 3.8% sodium citrate and standing in room temperature for an hour. Human blood samples were collected from three healthy volunteers by antecubital venipuncture in the morning after an 8-h fast. The protocol was approved Ethics Committee of Peking Union Medical College Hospital and the written informed consent was obtained from all the participating volunteers.

An *in vitro* flow model was assembled according to ASTMF1841 Standard (ASTM International, West Conshohocken, United States). The flow model was constructed with a circle of polyvinylchloride tube (0.5 mm in length and 3 mm in diameter), a peristaltic pump, with a water bath, and blood clots. A total of 15 ml normal saline was loaded as the liquid medium.

Because previous evidence demonstrated no significant thrombolytic enhancement with ultrasound or MBs alone, these control groups were not included in our experiment (Blinc et al., 1993; Nishioka et al., 1997; Hitchcock and Holland, 2010; Petit et al., 2012; Ammi et al., 2015; Engelberger et al., 2019). We weigh our efforts on minimizing the use of thrombolytic agents. Naturally, the idea of combined LFUS + MB treatment without thrombolytics prioritizes compared with conventional pharmacological treatment assisted by ultrasound. In the experiment, the blood clot was mounted in the flow tube. The blood clot was randomly subjected to no intervention, rhPro-UK infusion, intervention with LFUS plus MBs or crukMBs, namely the control, rhPro-UK, US + MB, and US + crukMB group with three repeats each. The treatment time was 30 min in all groups. In the rhPro-UK group, a bolus of 10 × 10^4^ U/kg rhPro-UK was infused into the test loop within 1 min. In the US + MB/US + crukMB group, the needle probe was inserted in the tube and imbedded in the clot, with ultrasound delivered for 30 min 1 ml MBs were infused to the treatment zone every 5 min (0, 5, 10, 15, 20, 25 min), with a total amount of 6 ml MBs delivered. The wet weight of blood clot before and after treatment was determined by an electronic balance.

### Rabbit inferior vena cava thrombosis model

The animal protocol followed the Guide for the Care and Use of Laboratory Animals (Eighth edition, National Academy of Sciences, United States) and was approved by the Animal Welfare and Ethics Committee of Peking Union Medical College Hospital. New Zealand white male rabbits (2.5–3.5 kg) were anesthetized with an intravenous injection of 20 mg/kg pentobarbital, followed by 10 mg at 30–60 min intervals through the auricular vein. Prior to the surgery, a bolus of penicillin (0.4 MU) was infused through the auricular vein for anti-infection prophylaxis. An approximately 6.0 cm midline laparotomy was performed with the rabbit in a supine position after removal of abdominal hair and sterilization of the surgical area. The colon and intestines were gently retracted aside, after which the retroperitoneum was opened and the inferior vena cava (IVC) was exposed. The approximately 2.0 cm vessel segment right distal to the left renal vein without any major branches was chosen to induce a thrombus. The blood flow was blocked by clipping the segment with two vascular clamps placed at the two ends of the selected segment. A bolus of thrombin (80 U, 0.1 ml) was injected into the IVC segment through a microinjector to produce a local thrombus. A bolus of heparin (200 IU/kg) was infused through the auricular vein 15 min after thrombus induction to prevent the further propagation of thrombus. After thrombus induction for 30 min, a loose ligation (vessel ligated together with an iron wire of 2.0 mm in diameter, followed by the removal of the iron wire) was performed at the proximal end of the thrombus to prevent the thrombus from dislodging, followed by the removal of vascular clamps and microinjector. The establishment of IVC thrombosis model was confirmed by ultrasonic evaluation. (Supplementary Figure S1).

### Thrombolysis *in vivo*

Following the establishment of IVC thrombosis model, rabbits were randomly assigned to four groups: the control, rhPro-UK, US + MB, and US + crukMB groups (*n* = 3 in the control and *n* = 6 in other groups). In the US + MB and US + crukMB groups, the needle transducer was inserted into the thrombus by piercing the vessel wall at the distal end of thrombus under the ultrasonic guidance (Figure 1B, Supplementary Figure S1)

In the rhPro-UK group, a bolus of rhPro-UK (3.3 × 10^4^ U/kg) in 6 ml was infused through the left auricular vein, the bolus infusion was completed within a minute. After the rhPro-UK injection, ultrasonic evaluation of thrombolysis was performed in a 5-min interval for 30 min, and in a 15-min interval for another 90 min. For the control group, a bolus of 6 ml of 0.9% (w/v) saline was infused with the same rate while the thrombus was left untreated.

For the US + MB and US + crukMB groups, the treatment protocol was the same as described *in vitro*. Ultrasonic evaluation of sonothrombolysis was evaluated in a 5-min interval for 30 min.

### Ultrasonic evaluation

Ultrasonic evaluation *in vivo* was performed using a portable diagnostic US system with a i12L-RS probe (mechanical index [MI], 0.5; frequency 10.0 MHz). During the treatment, the longitudinal and cross-sectional view of the thrombus were imaged using B-mode. The thrombus area was calculated using ImageJ software. Residual thrombus was evaluated by the ratio of thrombus area after and before the treatment. The BFV at the occlusion site was monitored using color flow mode and pulse-wave Doppler. Each BFV value was the average of five measurements.

### Safety and pathologic evaluation

In the *in vitro* study, for histological examination, thrombus sample was subjected to H&E staining and SEM.

In the *in vivo* study, all rabbits were euthanized with a lethal dose of pentobarbital (100 mg/kg) 24 h after treatment. The IVC segment at the treatment zone was excised, and were fixed in 10% formaldehyde solution for 24 h and were embedded in paraffin. Consecutive sections in the middle part of the thrombus in the IVC were stained with H&E, MSB, and an anti-fibrinogen antibody to observe histological changes in the cross-sectional area of the thrombus.

Various organs (heart, lungs, liver, spleen, kidneys, brain) were collected for gross examination and histological evaluation from three randomly selected rabbits in each group. Possible intracranial hemorrhage was evaluated under gross and microscopic observation. Detailed results of intracranial hemorrhage examination were shown in the Supplementary Table S1 and Supplementary Figure S5.

Hematological specimens were collected and sampled for a series of laboratory testing. To evaluate the possible organ and tissue damage of the treatment, hematological specimens were collected from another auricular vein into a 2.0 ml vacuum blood collection tubes pre- and post-treatment (*n* = 3–5). A series of laboratory values (alanine aminotransferase, ALT; lactate dehydrogenase, LDH; blood urea nitrogen, BUN; serum creatinine, Cr) were determined within 30 min using an automated analyzer.

The thermal effect of sonothrombolysis was monitored in five rabbits at the end of each round of treatment by placing the probe of thermometer at the interspace between the posterior wall of IVC and posterior abdominal wall.

### Statistical analysis

SPSS 25.0 software (IBM, New York, United States) was used for statistical analyses. Results were given as mean value ± standard deviation. Multiple comparisons were performed using 1-way ANOVA with the Bonferroni multiple comparison test. Comparisons between two groups were performed using student’s t tests. A *p*-value of < 0.05 was considered to be statistically significant.

## Data Availability

The original contributions presented in the study are included in the article/Supplementary Material, further inquiries can be directed to the corresponding authors.

## Abbreviations

BFV, blood flow velocity; bMB, blank microbubble; CREKA, Cys-Arg-Glu-Lys-Ala peptide; creMB, CREKA microbubble; crukMB: CREKA/rhPro-UK microbubble; FITC, Fluorescein Isothiocyanate; H&E, hematoxylin and eosin; IHC, immunohistochemical; I_SPTA_, intensity spatial peak temporal average; IVC, inferior vena cava; LR, lysis rate; MB, microbubble; MI, mechanical index; MSB, Martius scarlet blue; PTS, post-thrombotic syndrome; RGDS, Arg-Gly-Asp-Ser peptide; rgdMB, RGDS microbubble; rhPro-UK, recombinant human prourokinase; SEM, scanning electron microscopy; TAMRA, Carboxytetramethylrhodamine; US, ultrasound; VLR, velocity of clot lysis; VTE, venous thromboembolism.
