# Supplementary material for: Enhanced thrombolysis by endovascular low-frequency ultrasound with bifunctional microbubbles in venous thrombosis: in vitro and in vivo study
Source: Front Bioeng Biotechnol. 2022 Jul 22;10:965769. doi: 10.3389/fbioe.2022.965769 (PMC9356075; doi:10.3389/fbioe.2022.965769)
Supplement: Supplementary file 2 [file DataSheet1.docx]

**Enhanced Thrombolysis by Endovascular Low-frequency Ultrasound with Bifunctional Microbubbles in Venous Thrombosis: in vitro and in vivo study**

Zhaojian Wang^1^, Yunfan Pan^2^, Huaigu Huang^1^, Yuan Zhang^3^, Yan Li^2^, Chenghong Zou^3^, Guanghua Huang^1^, Yongjian Li^2^, Jiang Li^3*^, Haosheng Chen^2*^, Yuexin Chen^1*^.

**Materials and Methods**

**Preparation and characterization of crukMBs**

Commercially available phospholipid-encapsulated sulfur hexafluoride MBs (SonoVue, Bracco Imaging, Switzerland) were prepared according to manufacturer’s instruction. For the preparation of fibrin-targeted CREKA/rhPro-UK MBs (crukMBs), 0.2mg of Cys-Arg-Glu-Lys-Ala peptide (CREKA, Qiangyao Biotechnology, China) and 5×10^5^U of recombinant human prourokinase (rhPro-UK, Tasly Biopharmaceuticals, China) was mixed with 5ml of suspension of MBs for 10 minutes with a vortex shaker (DLAB MX-RD-E, Medilab Tech, USA) at the speed no more than 80 rpm. Next, the sample was centrifuged (Eppendorf, MiniSpin, USA) at 800rpm (40g) for 2 minutes. The undernatant was discarded and replenished with normal saline. All the MB solutions were diluted to a fixed volume of 6 ml before treatment. For the fluorescent double labeling, CREKA was labeled by FITC showing green light, and rhPro-UK were labeled by 5-TAMRA showing red light. MBs modified with only CREKA peptide were defined as creMBs. Same amount of Arg-Gly-Asp-Ser peptide (RGDS, Qiangyao Biotechnology, China) were added under the same protocol to make rgdMBs.

Concentration and size distribution of the MBs were determined using Image Particle Size Analyzer (Pixtreme, W2000M, USA). Analysis was performed by adding 1ml of the sample to 1.5 mL 0.9% normal saline and pushing the liquid into the sampling area with a syringe. The particle size of microbubble sample is automatically analyzed by the software system, and the concentration needs to be calculated simply according to the pixel size of X/Y axis (field of view pixel: 3840×2748, X direction: 0.1579 μm/pixel, Y direction: 0.1579 μm/pixel) and the height (100μm) of the liquid pool to obtain the effective volume (V/μm^3^) of a single photo. Divide the total number of particles (N) of the sampled photos (n) counted by the software by the effective volume of a single photo and number of photos, and then multiply by the dilution multiple (×2.5) to obtain the particle concentration (c/MBs· ml^−1^), as shown in formula (S1).

$c=\frac{2.5*N}{V*n}=\frac{2.5*N}{3840*0.1579*2748*0.1579*n}\left（ \frac{MBs}{{\mu m}^{3}} \right）=\frac{2.5*N}{263094.7686912*n}*{10}^{12}（MBs/ml）$*(S1)*

Drug loading capacity of microbubbles was determined by Bradford protein assay kit (Beyotime Biotechnology, China). In brief, rhPro-UK loaded MBs were ruptured prior to the assay. 10μl samples were dripped to the well of 96-well plate (Corning, USA) with 300μl staining solution. The concentration of rhPro-UK was determined by correlating the absorbance at 595 nm with a microplate reader (Infinite, USA) to the standard curve.

Adhesion analysis was performed on the clot smear. The clot smear was prepared by applying 2.5μl of the mixture of plasma and coagulant (75 mM CaCl_2_, 35 mM MgCl_2_ in PBS) in a volume ratio of 1:9 to the wells of 96-well plate. The mixture was evenly smeared to form a thin layer and incubated at room temperature for 30 minutes. A total of 100μl MBs were added to each well with 200μl 0.9% normal saline. The plates were then sealed with sealing film and inverted for 10 minutes to ensure the full exposure of MBs to the surface of the clot smear. After that, the plates were inverted back for 10 minutes. The sealing film was removed and 200μl upper liquid was discarded. Next, the number of MBs attached to the clot smear was analyzed under the microscope (Olympus IX83, Japan). (Figure S6)

**In silico simulation of oscillation of microbubbles**

The characteristics of microbubbles oscillation varies as the parameters of external ultrasound field changes. Oscillations of microbubbles are simulated with MATLAB R2020b, using the microbubble oscillation model developed by Marmottant et al.^[1]^ The formulas and parameters are as follows.

$$\rho_{l}\left( R\ddot{R}+\frac{3}{2}\dot{R}^{2} \right)=\left( p_{0}+\frac{2\sigma\left( R_{0} \right)}{R_{0}} \right)\left( \frac{R}{R_{0}} \right)^{-3\kappa}\left( 1-\frac{-3\kappa}{c}\dot{R} \right)-\frac{2\sigma\left( R_{0} \right)}{R}-\frac{4\mu\dot{R}}{R}-\frac{4\kappa_{s}\dot{R}}{R^{2}}-p_{0}-p_{\mathrm{ac}}(t)$$

$R$: the radius of the bubble wall

$\dot{R}$: the velocity of the bubble wall

$\ddot{R}$: the acceleration of the bubble walls

$\rho_{l}$: the density of the liquid

$p_{0}$: the ambient pressure

$\sigma\left( R \right):$ the surface tension, a function of the radius R, $\sigma\left( R \right)=\chi(\frac{R^{2}}{{R_{0}}^{2}}-1)$, where $\chi= 0.55 N/m$ and $\kappa_{s}= 2.3\times{10}^{-8}kg/s$ for Sonovue.

$\kappa$: the polytropic gas exponent

$\mu$: the viscosity of the surrounding water

$c$: the speed of sound

$p_{\mathrm{ac}}(t)$: the applied acoustic field, a function of time t, $p_{\mathrm{ac}}\left( t \right)=PNP\sin(2\pi ft)$, where PNP is the peak negative pressure of the acoustic field, and f is the frequency.

Microbubble oscillation under acoustic fields with a PNP of 50kPa and a frequency of respectively 2MHz (high frequency ultrasound typical for medical use) and 47.1kHz (LFUS used in this study) are simulated.

**Pathological evaluation**

After the experiment, the rabbits were sacrificed through a lethal dose of pentobarbital. Various organs (heart, lungs, liver, spleen, kidneys, brain) were collected for gross examination and histological evaluation from 3 randomly selected rabbits in each group. Possible intracranial hemorrhage was evaluated under gross and microscopic observation. The whole brain harvested was frozen in -20℃ for 1 hour, then were coronally sliced at an interval of 3.0 mm. A total of 10-12 slices were obtained and evaluated. Parenchymatous intracerebral hemorrhages, and punctuate hemorrhages or isolated small red marks within the tissue were identified and examined by microscopic observation after H&E staining. Parenchymatous intracerebral hemorrhage is defined by the evidence of hemorrhages in parenchymatous of brain with the red speckling of an area that cannot be washed off by normal saline. Punctuate hemorrhage is defined by isolated extravascular accumulation of red blood cells within the brain tissue under the microscopy.

**
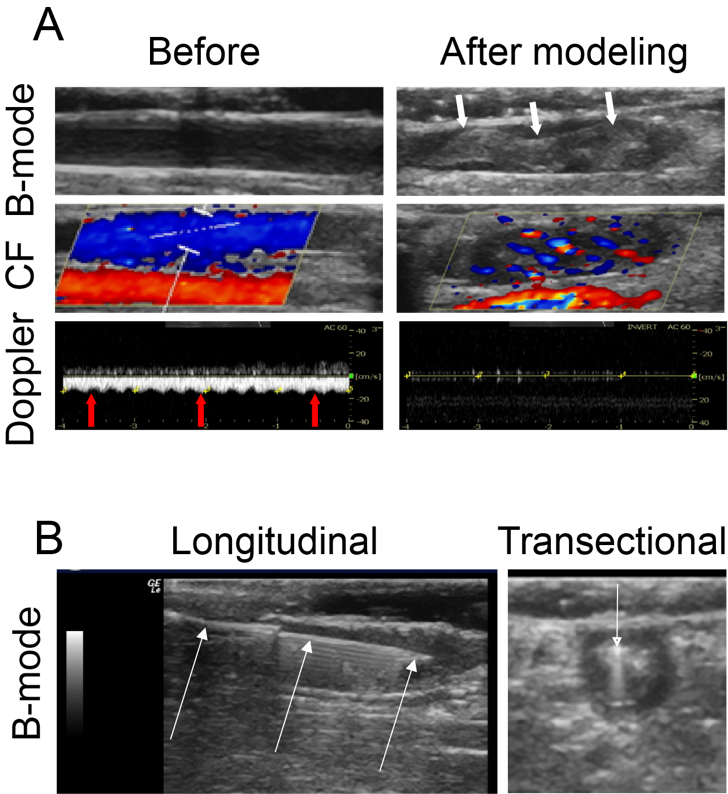
**

Figure S1 Establishment of rabbit IVC thrombosis model. The blood clot in the IVC was confirmed by ultrasonic evaluation(A). Mean blood flow velocity (BFV) before and after the induction of IVC thrombus were 12.14±3.43 cm/s, 0.20±0.29 cm/s, respectively, indicating the successful establishment of rabbit IVC thrombosis model. After the model establishment, the needle probe (thin white arrow) was inserted into the thrombus to perform sonothrombolysis (B).

**
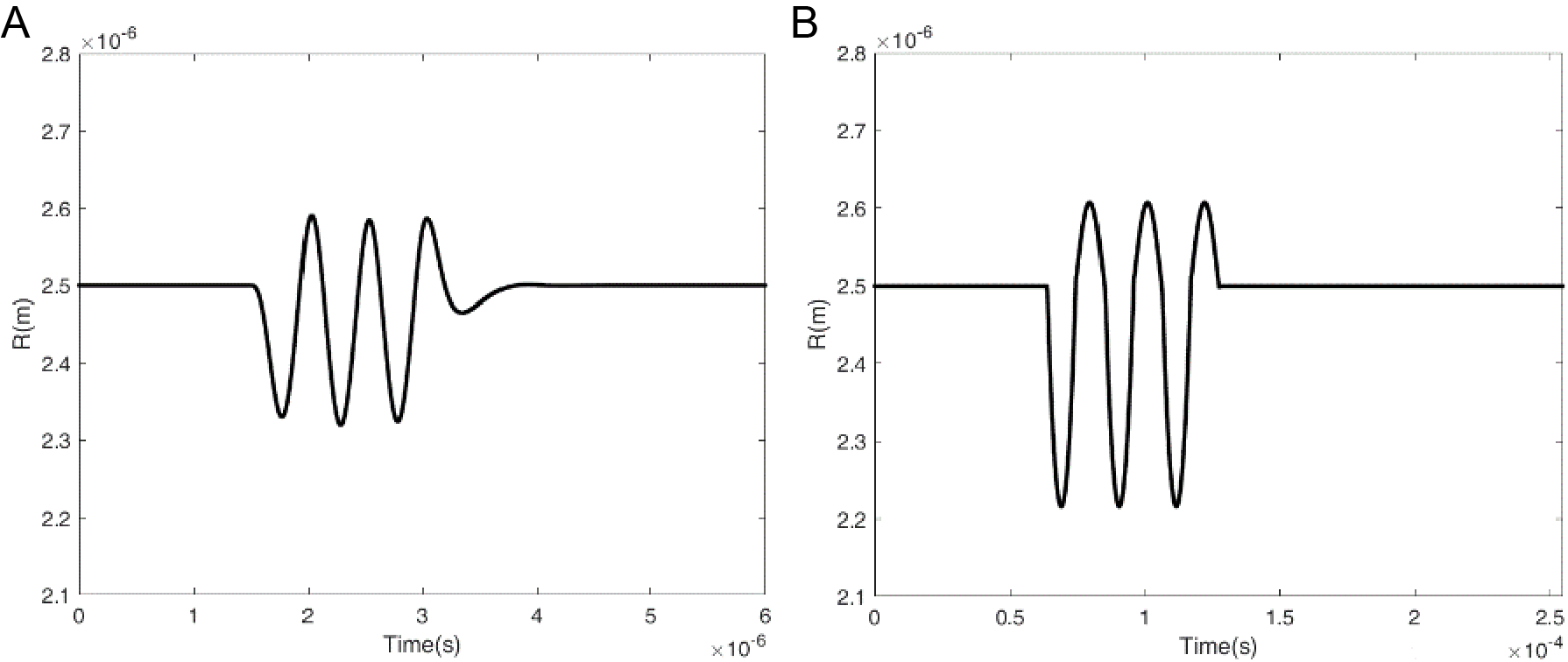
**

Figure S2. Oscillation of a 2.5μm microbubble at 2 MHz in plasma (A). Oscillation of a 2.5μm microbubble at 47.1kHz in plasma (B).

**
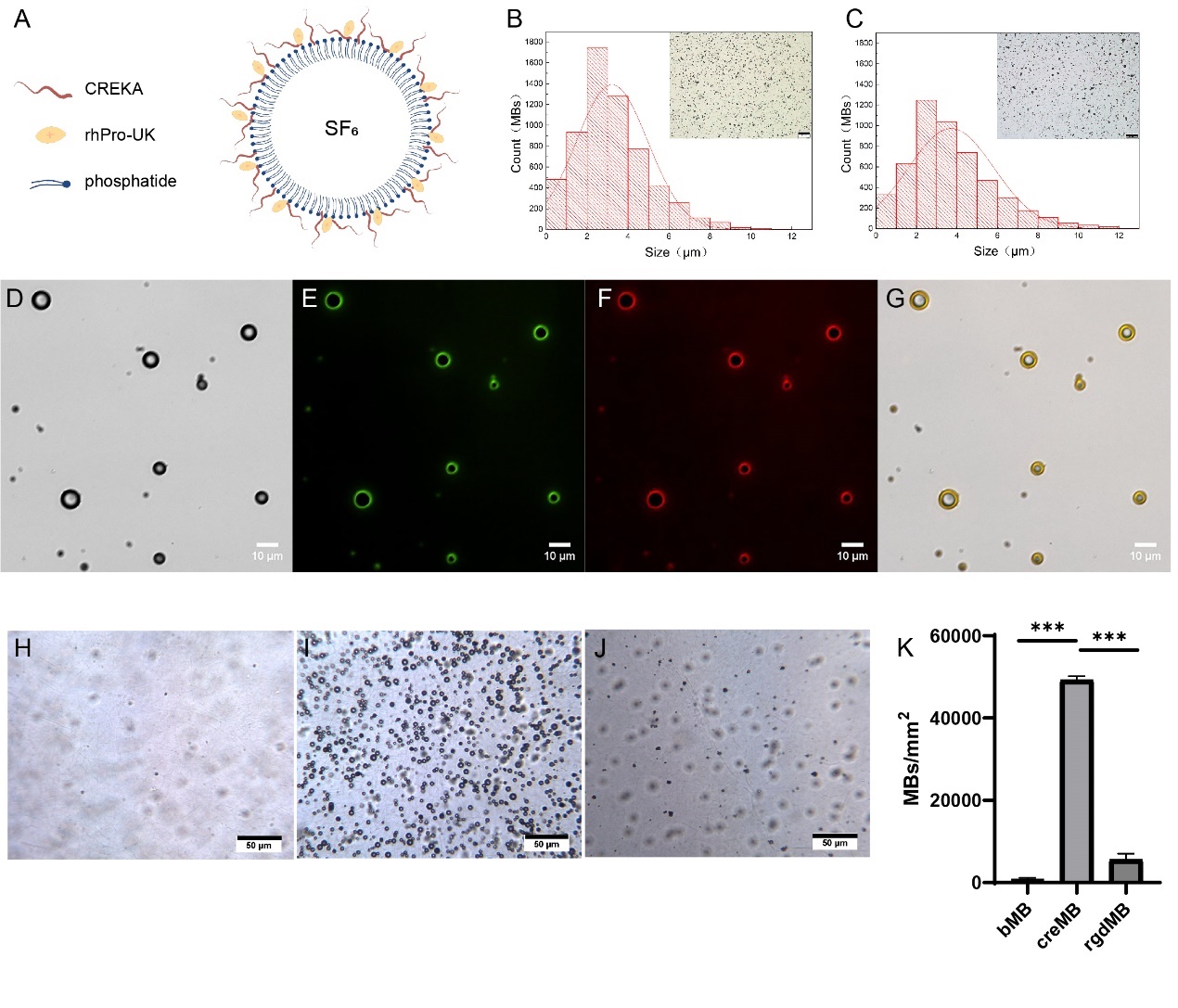
**Figure S3. Characterization of CREKA/rhPro-UK MBs. Schematic representation of crukMB (A). The size distributions of blank MBs(bMB) (B) and crukMBs (C) were similar. Only a negligible number of microbubbles have the diameters greater than that of erythrocytes, indicating that gas emboli are highly unlikely. (B, C; scale bars, 50μm). A ring-shaped luminance was confirmed under bright-field and fluorescent microscopy, indicating that the surface of crukMBs was coated by the thrombolytic agent rhPro-UK and the targeting ligand CREKA peptide (Figure 3D to 3G, scale bars, 10μm). Further, the fibrin-based targeting strategy was compared with the platelet-based targeting strategy using RGDS peptide. RGDS peptide targets the GP IIb/IIIa receptors on activated platelets. ^[2]^Adhesion analysis was performed to compare the targeting ability of the CREKA-MBs(creMBs) and RGDS-MBs (rgdMBs). A significantly higher density of MBs was observed in the creMBs(I) compared with rgdMBs(J) and bMBs(H) (4.92±0.09×10^4^ versus 0.56±0.15×10^4^ MBs/mm^2^, and 0.76±0.42×10^3^ MBs/mm^2^, respectively, scale bars, 50μm, ***p<0.001) (K), indicating that the fibrin-based targeting strategy is superior to the platelet-based targeting strategy for the targeting of fibrin-rich blood clots.


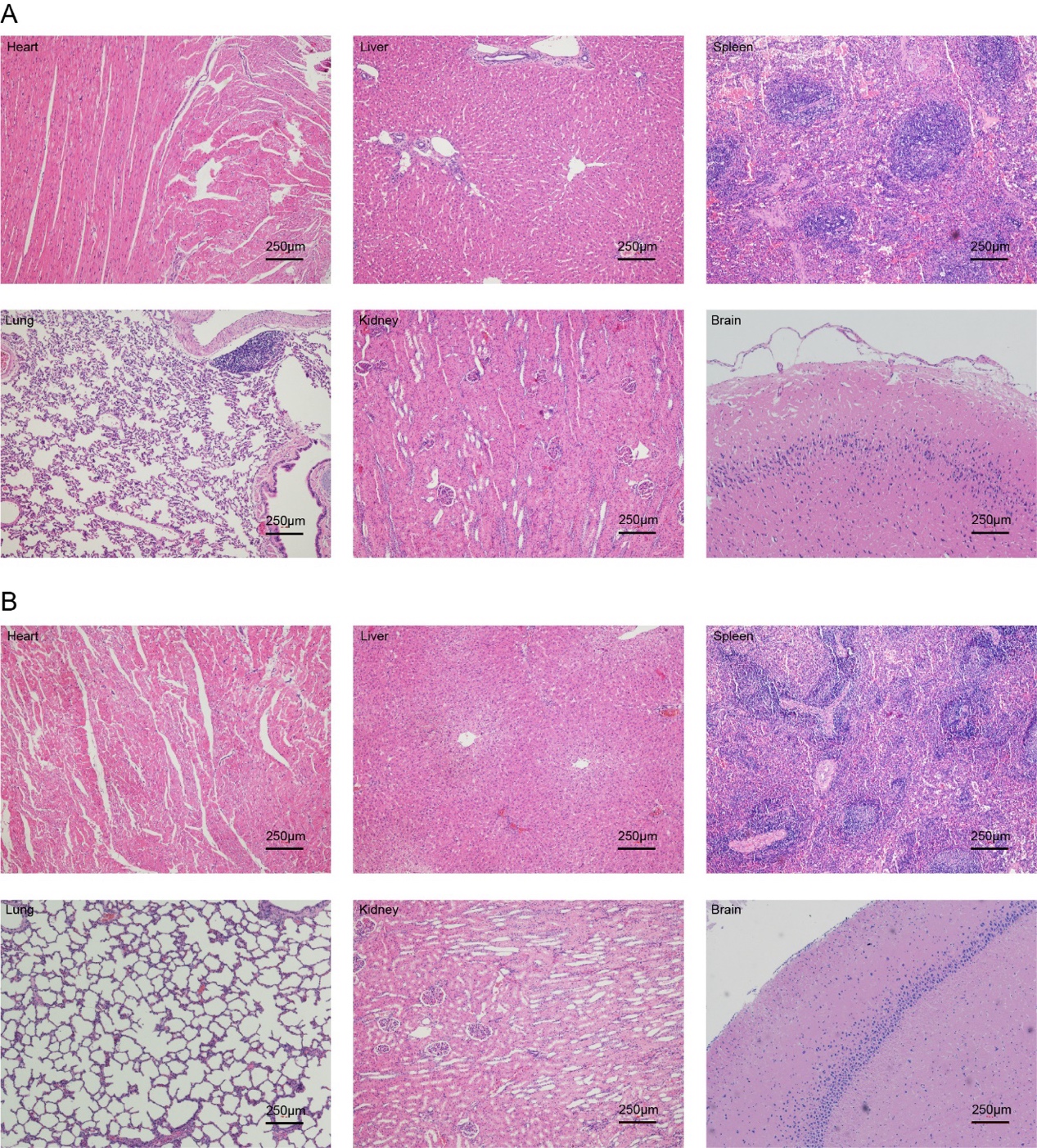


Figure S4. Representative H&E staining of major organ samples from rabbits after treatment with control (A) or US+crukMB group (B). The stained images show no obvious injury or necrosis (i.e., little swelling and few inflammatory cells, such as neutrophils, and apoptotic cells) in the IVC thrombosis models.


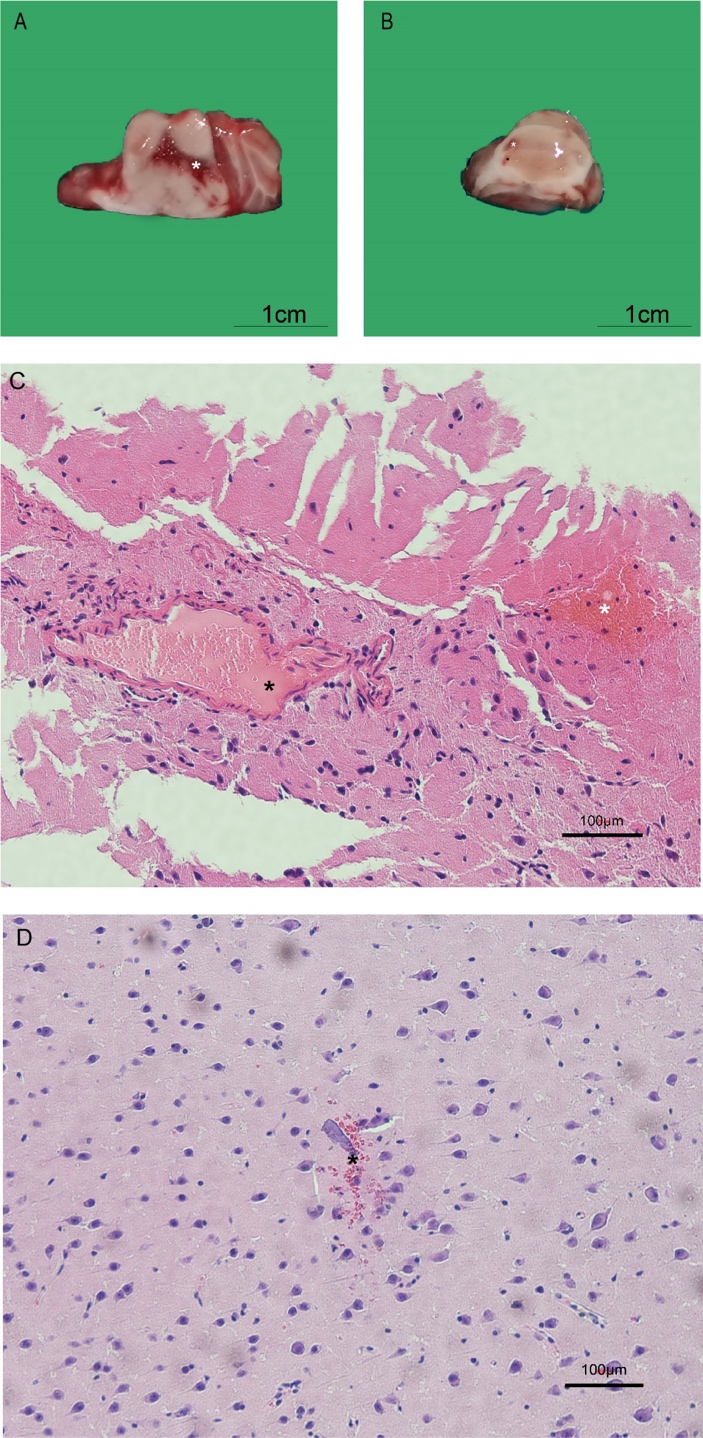


Figure S5. Records of punctuate hemorrhages under microscopy and grossly identified parenchymatous intracerebral hemorrhages. Intracerebral hemorrhages were identified in one rabbit in the US+bMB group (A, indicated by white asterisk ) and in one rabbit in the rhPro-UK group (B, two hemorrhage specklings indicated by white and black asterisks). Two punctuate hemorrhages were identified, in the US+bMB group (C) and rhPro-UK group (D) respectively. Note that the black asterisk in C is not hemorrhagic site because the red blood cells and fibrin are limited within the vessels. While the white asterisk in C and black asterisk in D indicate the punctuate hemorrhages. These findings are thought to be opportunistic. No difference in intracerebral hemorrhage is indicated between the groups.

Figure S6. The difference of adhesion between microbubbles and thin layer thrombus. SonoVue-CREKA-rhProUK adhered to the thrombus surface (A) SonoVue adhered to the thrombus surface (B). Background denoising of figure A (C) Background denoising of figure B (D)


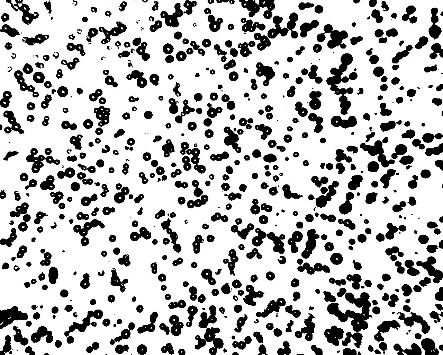

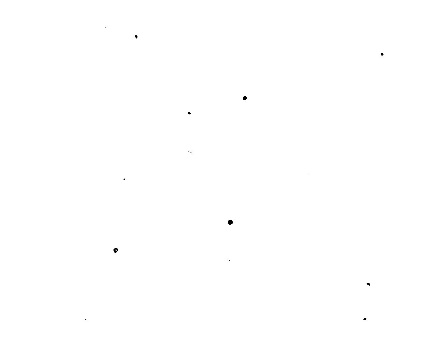

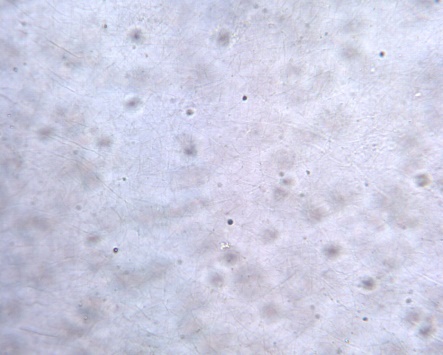

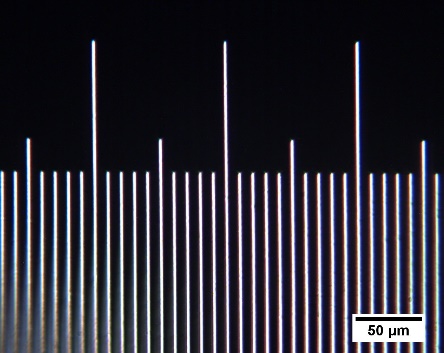

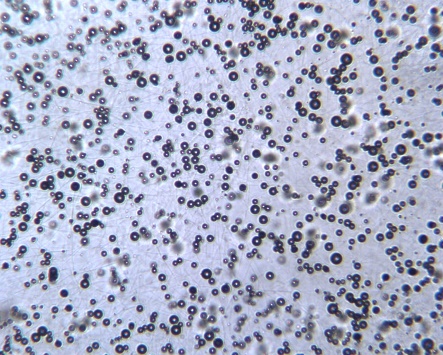

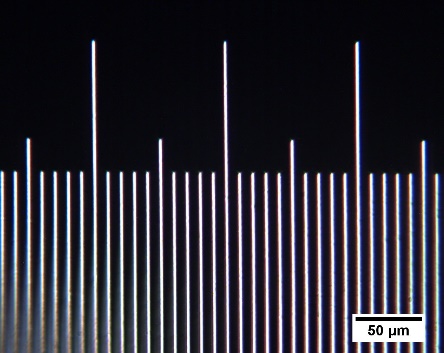


**B**

**C**

**A**

**D**

| **Group** | **Number of punctuate hemorrhages under microscopy** | **Number of parenchymatous intracerebral hemorrhages under gross examination** |
| --- | --- | --- |
| Control (n=3) | 0 | 0 |
| rhPro-UK (n=6) | 1 | 1 |
| US+bMB (n=6) | 1 | 1 |
| US+crukMB (n=4) | 0 | 0 |

**Table S1** Number of parenchymatous intracerebral hemorrhages and punctuate hemorrhages under microscopy.

[1] MARMOTTANT P, MEER S V D, EMMER M, et al. A model for large amplitude oscillations of coated bubbles accounting for buckling and rupture [J]. J Acoust Soc Am, 2005, 118(6): 3499-505.

[2] WANG Z, HUANG H, CHEN Y, et al. Current Strategies for Microbubble-Based Thrombus Targeting: Activation-Specific Epitopes and Small Molecular Ligands [J]. Frontiers in bioengineering and biotechnology, 2021, 9: 699450.
